# A novel cell senescence-related IncRNA survival model associated with the tumor immune environment in colorectal cancer

**DOI:** 10.3389/fimmu.2022.1019764

**Published:** 2022-10-06

**Authors:** Chengfei Xu, Fanghan Li, Zilin Liu, Chuanjing Yan, Jiangwei Xiao

**Affiliations:** ^1^ Department of Gastrointestinal Surgery, The First Affiliated Hospital of Chengdu Medical College, Chengdu Medical College, Chengdu, China; ^2^ School of Clinical Medicine, Chengdu Medical College, Chengdu, China

**Keywords:** long non-coding RNAs(lncRNAs), colorectal cancer, immune cell infiltrate, cell senescence, survival analysis, survival model.

## Abstract

Long noncoding RNAs have a major role in tumorigenesis, development, and metastasis in colorectal cancer (CRC), participate in the regulation of cell senescence and are related to the prognosis of CRC. Therefore, it is important to validate cell senescence-related lncRNAs that correlate with prognosis in CRC.

**Methods:**

CRC expression profile data and clinical information were downloaded from TCGA. A gene list related to cellular senescence was obtained from Human Aging Genomic Resources. A coexpression network of cell senescence-related mRNA−lncRNA was explored with R. Six cell senescence-related lncRNA signatures were identified by univariate and multivariate analyses. The cell senescence-related risk model was generated by using six cell senescence-related lncRNAs, and the risk score was calculated. Furthermore, an internal validation set and GSE17537 were used to verify the risk model. The risk model demonstrated good stability and accuracy. Finally, we investigated the correlation between cell senescence-related risk scores and immune infiltration, immune function, immune checkpoints, and drug sensitivity.

**Result:**

We established a signature of six cell senescence-related lncRNAs. The cell senescence-related risk model revealed an exceptional ability to assess the prognosis of colorectal cancer and was correlated with clinical features. Additionally, we observed that risk models correlate with the tumor microenvironment and immune checkpoints, potentially predicting patient response to clinical immunotherapy. Finally, we validated the correlation between the cell senescence-related risk model and drug susceptibility. Our findings indicated that AICAR, cisplatin, nilotinib, and bexarotene exhibited lower IC50 values in the high-risk group.

**Conclusion:**

Our current study identified 6 cell senescence-associated lncRNA signatures that may be vital biomarkers to predict the prognostic features and immune and chemotherapy responses in CRC.

## Background

Colorectal cancer (CRC), which accounts for more than 90% of colorectal carcinomas, ranks as the third most diagnosed malignant tumor and the second most common cause of death in men and women (1, 2). In 2020, the number of newly diagnosed patients with CRC was more than 1.9 million, and the number of deaths was 935,000 (1). Multiple pervasive factors may drive CRC, including aberrant gut flora obesity, positive family history, a Western lifestyle, unhealthy behaviors, inflammatory bowel disease, and smoking (3–6). Moreover, several studies have shown that aging may be strongly associated with CRC (6–8). Statistics have demonstrated that the incidence of CRC tends to be younger (9). However, it is still dominated by new cases in middle-aged and elderly people, and the incidence increases year by year with age (10, 11). Before the age of 50, the incidence rate approximately doubles for every 5 years of age, and after the age of 55, the incidence rate increases by approximately 30% every 5 years (11). Currently, the treatment of CRC is mainly based on AJCC staging in the United States (12). The treatment effect of CRC is still poor, although there are many treatment methods, including surgery, chemotherapy, immune treatment, and targeted therapy (13). If distant metastasis occurs, the 5-year overall survival (OS) is lower than 14% (14). Patients with the same AJCC stage have different therapeutic effects to the same treatment. Therefore, it is crucial to explore novel prognostic biomarkers and promising targets.

With the aging of populations worldwide and increased life expectancy globally, rapid growth in the elderly population will lead to an unprecedented increase in cancer cases and deaths (15). In recent decades, researchers have suggested that the relationship between aging and tumors is very close (16, 17). Cell senescence is one of the characteristics of aging, and it can promote aging through various mechanisms. In the latest 3rd edition of cancer hallmarks proposed in 2022, 4 novel members have been joined, and one of the hallmarks is senescent cells (18). Cellular senescence plays a vital role in tumors. Oncogenes can induce cell senescence. S Courtois-Cox et al. showed that upregulating oncogenic HRAS^G12V^ led to permanent cell cycle arrest (19). Expression of BRAFV600E can induce cell senescence and apoptosis by inhibiting the BRAF-MEK-ERK pathway by upregulating IGFBP7 (20). Second, senescent cells can shape the tumor microenvironment (TME) through the senescence-associated secretory phenotype (SASP). It can recruit and activate immune cells to exert an antitumor effect and can also promote tumor cell proliferation (21). Therefore, SASP is a double-edged sword. For example, overexpression of IL8RB can lead to cellular senescence through a p53-dependent mechanism (22). Currently, there are many studies on cell senescence in CRC (23–25). Thus, it is important to explore the key cell senescence-related signature with prognostic significance in patients with CRC.

In the past decade, lncRNAs, which are noncoding transcripts of 200 nucleotides in length, have been shown to be involved in tumorigenesis, development, and metastasis. Mounting evidence has shown that lncRNAs have an intimate relationship with cell senescence. For example, recent findings indicate that lncRNA SNHG12 can protect cells from cell senescence *via* a DNA-PK-mediated DNA damage response (26). In recent years, an increasing number of studies have used lncRNAs to construct diagnostic and prognostic models for gastric cancer, rectal cancer, and hepatocellular carcinoma (27–29). There are currently no studies on clinical prognostic models of cell senescence-related lncRNAs. Therefore, our study used cell senescence-related lncRNAs to construct a risk model.

## Materials and methods

### Download the data and extract the clinical information

**Figure 1:**
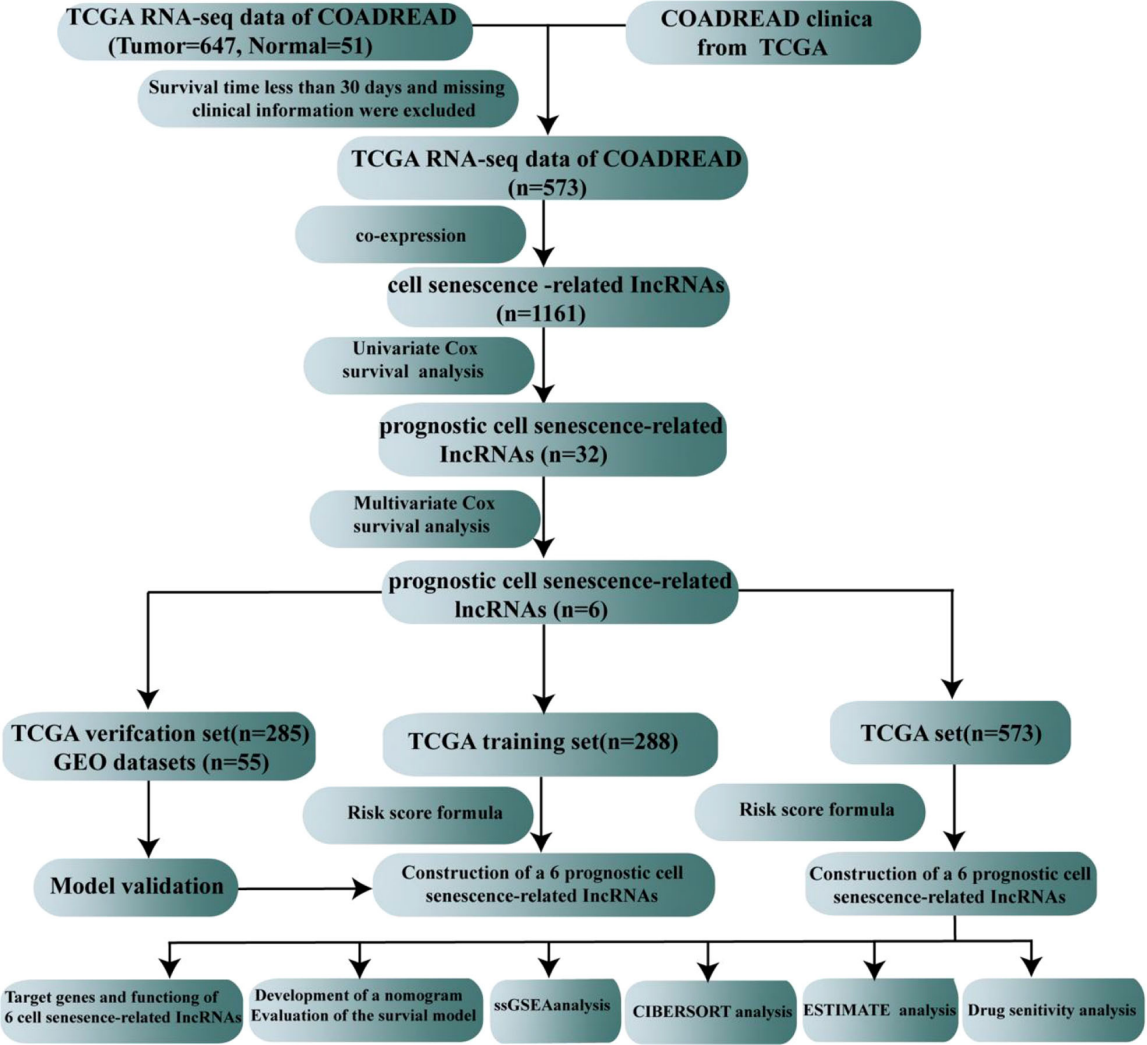
The flow sketch of our study.

The flow sketch of our research is demonstrated in **Figure 1**. The raw gene expression data and the clinical characteristics of colorectal cancer patients were obtained from TCGA (https://portal.gdc.cancer.gov/), which included 647 colorectal carcinoma samples and 51 normal samples. The raw data were normalized by FPKM in R (version 4.2.1). We extracted clinical information on patients with CRC. Missing clinical features, including sex, age, stage, TNM stage, and overall survival, were excluded, and a total of 573 patients with CRC were used for our follow-up study. The GSE17537 dataset obtained from the Gene Expression Omnibus (GEO)https://www.ncbi.nlm.nih.gov/geo/) served as the external testing dataset.

### Extraction of cell senescence-related lncRNAs in CRC

A 279-cell senescence-related gene set was downloaded from Human Aging Genomic Resources (https://genomics.senescence.info/cells/). The limma package was loaded during our analysis (30). The cor.test function was used to correlate mRNA-lncRNA and calculate *Coef* and *p* values. Cell senescence-related lncRNAs were explored by establishing a cell senescence-related mRNA−lncRNA relationship based on the cutoff value of |*Coef*| > 0.4 and *P*<.001.

### Identification of cell senescence-related lncRNA prognostic signatures from colorectal malignant tumors

To explore the cell senescence-related lncRNAs linked with survival status, we adopted univariate Cox regression survival analysis with the ‘Survival’ package and Kaplan−Meier analysis. Furthermore, to screen out promising OS-associated lncRNAs, we screened these cell senescence-related lncRNAs linked with survival status by multivariate CPHR analysis (stepwise model). we employed the Akaike information criterion (AIC) to obviate overfitting. OS-associated lncRNAs with the highest likelihood ratios and lowest AIC scores were identified from the stepwise model. The risk score formula was in accordance with the formula below:


$$risk\;score(RS)=\sum_{i=1}^{n}\left(Coef_{i}\times x_{i}\right)$$

*Coef* represents the coefficient value, and x represents the expression value of selected cell senescence-related lncRNAs. The dataset of colorectal malignant tumors was classified into two datasets (the training set and the validation set) with a randomized approach. We used the median risk score as a cutoff point, and the training set was divided into two groups (a high-risk group and a low-risk group).

### Internal validation set and GSE17537 to validate the risk model

The TCGA-COADREAD internal validation set and GSE17537 external data validation were used for risk score validation. The samples in the internal cohort and external validation group were classified into high-risk and low-risk groups based on the risk scores calculated by the risk formula. Differences in OS between the two subgroups were explored using Kaplan−Meier survival analysis. To further explore the accuracy of the risk model, AUC points for 1, 3, and 5 years in the ROC curves were generated.

### Target gene and function analysis of the 6 cell senescence-associated lncRNAs

ENCORI (The Encyclopedia of RNA Interactomes) is an openly licensed, state-of-the-art platform (https://starbase.sysu.edu.cn) that can be utilized to explore RNA-binding protein (RBP) and RNA interactions, RNA−RNA interactions, and their potential functions and mechanisms in human disease (31). We used ENCORI’s ceRNA method to screen target genes of 6 cell senescence-associated lncRNAs with a cutoff value of miRNA number ≥ 2, *P value* ≤ 0.01 and FDR value ≤ 0.01. The ‘clusterProfiler’ package and the ‘enrichplot’ package were used to perform KEGG and GO enrichment analyses of target genes in R.

### Identification of a nomogram and evaluation of the survival model

We constructed a nomogram that predicted 1-, 3-, and 5-year survival by combining the risk score in R. A calibration curve was used to evaluate whether the survival estimation was consistent with the actual survival rate. Next, to assess whether the risk model is an independent prognostic indicator for CRC, we employed univariate and multivariate Cox regression analyses. PCA is a commonly used dimensionality reduction tool in the field of computer vision (32). We employed the’scatterplot3d’ package to evaluate possible discrepancy between the high- and low-risk groups. Finally, we validated the relationship of risk scores and 6 cell senescence-associated lncRNAs in various clinical features, including age, sex, T stage, N stage, M stage, and stage.

### Analysis of ssGSEA, CIBERSORT, ESTIMATE and immune checkpoint

Immune Function and Immune Cell Files The ‘immune.gmt’ was obtained from the ssGSEA website (http://www.gsea-msigdb.org/gsea/index.jsp) (33). The gene expression data of different immune functions and immune cells were converted into scoring data by the ‘GSVA’ package in R. The risk file was merged with the immunization file. A Wilcoxon test was used for comparison.

CIBERSORT is based on a deconvolution algorithm used to calculate the infiltrating degrees of 22 immunocyte types. The source code of the LM22 eigengene matrix sequence was downloaded from the cibersortx website (https://cibersortx.stanford.edu). The infiltration rates of 22 immune cell types were identified using CIBERSORT R script v1.03. The calculated relative infiltration rate was merged with the risk score. A Wilcoxon test was used for comparison.

Estimation of stromal and immune cells in malignant tumor tissues using expression data (ESTIMATE) (34) utilizes the transcription of cancer samples to infer the content of tumor cells, as well as infiltrated immune cells and stromal cells. The proportion or abundance of immune cells, stromal cells, and tumor cells in tumor tissue associated with the tumor microenvironment can be calculated. Each sample was scored with the ESTIMATE package in R. A Wilcoxon test was used for comparison.

Immune checkpoints belong to a variety of molecules expressed on immune cells that can adjust the degree of immune activation. They have a major role in limiting excessive immune activation. Immune checkpoint genes were extracted from the literature. Genes for immune checkpoints were extracted from the literature (35–39). The differential gene expression values extracted from the expression profile file were merged with the risk score. A Wilcoxon test was used for comparison.

### Drug sensitivity analysis

According to Cancer Drug Susceptibility Genomics (GDSC) (https://www.cancerrxgene.org/), which is the largest openly available pharmacogenomics database, drug responses to each sample are predicted based on the transcriptome of the sample (40). Medication data processing was explored by the ‘pRRophetic’ package (41). We used regression to obtain IC50 estimates for specific chemotherapy drug treatments and used 10 cross-validations to measure the accuracy of regression and prediction for the GDSC training set. Default values were chosen for all parameters, including “battle” to remove batch effects and duplicate gene expression averages. Finally, we used GDSC to predict the IC50 and AUC of drugs in CRC cell lines.

### Statistical analysis

All the data manipulation and statistical analyses were carried out using R software. (version 4.2.1). The mRNA−lncRNA relationships associated with cell senescence were analyzed by Pearson correlation with cutoff points set at |Coef| > 0.4 and *P*<0.001. We employed univariate and multivariate Cox regression analyses to determine cell senescence-related lncRNAs as independent prognostic factors for CRC. Survival curves in the current research were created by the Kaplan−Meier approach and tested by log-rank. ROC analysis was used to measure prognostic risk scores. Immune function, immune cells, immune checkpoints, and immune infiltration were assessed using Wilcoxon tests. The R code we used to analyze is shown in **Supplementary Code 1**.

## Results

### Investigation of cell senescence-related lncRNAs in CRC

**Figure 2:**
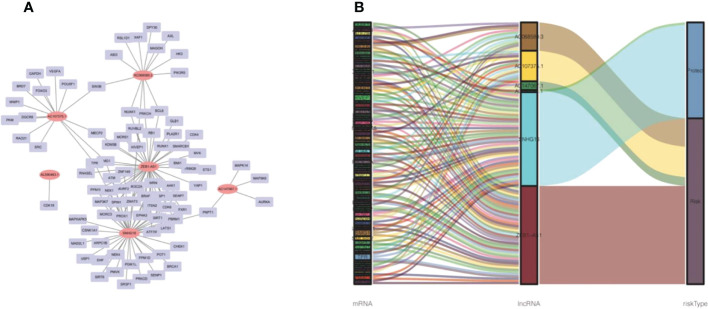
Prognostic cell senescence‐related mRNA and lncRNA coexpression network in CRC. **(A)** A coexpression network of the 6 cell senescence‐related lncRNA‐mRNAs. **(B)** Sankey diagram of mRNAs, lncRNAs, and risk type.

We established 1,161 cell senescence-related lncRNAs in TCGA-COADREAD based on the coexpression relationship between cell senescence-related genes and cell senescence-related lncRNAs (**Supplementary Table S1**). Univariate Cox regression analysis demonstrated that 32 cell senescence-related lncRNAs were screened (*P* and K-M< 0.05), including 4 lncRNAs with protective factors and 28 lncRNAs with risk factors (**Table 1**). Furthermore, the 32 lncRNAs were enrolled in a multivariate Cox regression analysis to identify 6 genes as signature genes, including 4 lncRNAs with protective factors and 2 lncRNAs with risk factors (**Table 2**). We visualized the cell senescence-related lncRNA−mRNA coexpression network (**Figures 2A, B**).

**Table 1:** Univariate Cox regression analyses and K-M analysis in patients with CRC.

	K-M analysis	Univariate cox regression analyses			
lncRNA	K-M *P value*	HR	HR 95 low	HR 95 high	*P value*
AC083843.2	0.025982261	1.072251827	1.000752882	1.148859026	0.04755325
SNHG16	0.005734488	0.851658227	0.760110643	0.954231786	0.005650684
LINC00957	0.016813225	1.328613198	1.062180914	1.661876057	0.012837841
AL139384.1	0.002699544	1.220156942	1.022296469	1.456312339	0.027507088
MIR4435-2HG	0.005331956	1.183229694	1.010016636	1.386147968	0.0372165
AC026471.4	0.029890747	1.117122274	1.027640228	1.214395993	0.009321878
LINC01836	0.008502524	1.141810566	1.015848943	1.283390978	0.026173522
AL590483.1	0.02763081	0.645481978	0.432166362	0.964089344	0.032465641
AC007383.2	0.038521548	1.189043095	1.04129153	1.357759514	0.01053856
AC027644.3	0.028738401	1.395998131	1.124925233	1.732391385	0.002456554
ZEB1-AS1	0.000817167	2.063247148	1.514987952	2.809915939	4.31E-06
AC107375.1	0.003808175	1.22604047	1.002723444	1.499092539	0.046980592
AC068580.3	0.011154045	1.316048225	1.055940598	1.640227616	0.014507309
BACE1-AS	0.046662051	1.150821388	1.018192373	1.300726565	0.024541438
NKILA	0.029750594	1.069281432	1.004198403	1.138582554	0.036552636
AC069281.2	0.004721843	1.39058977	1.144425015	1.689704334	0.000909589
AC073611.1	0.006737118	1.294672529	1.016641816	1.648738946	0.03628013
AC005261.3	0.002223117	1.18337291	1.017603156	1.376146915	0.028772813
LINC01011	0.007735977	1.392388183	1.049258382	1.847728725	0.021845154
AC105219.1	0.035665069	1.130766983	1.007022439	1.269717456	0.037681189
AC040977.1	0.000530753	1.175895785	1.056057622	1.309332813	0.003131605
AL161729.4	0.020038921	1.208589356	1.052496556	1.38783184	0.007250283
AL162586.1	0.002122635	1.385764325	1.133106901	1.694758687	0.00148939
AC011462.4	0.047639463	1.314572921	1.047087146	1.650389819	0.018454539
AC147067.1	0.047184163	1.170596096	1.026429655	1.335011331	0.018824389
LBX2-AS1	0.000606483	1.084244845	1.017598848	1.155255715	0.012455675
AC073896.3	0.020718137	0.584502965	0.370081243	0.923158693	0.021287921
AC093673.1	0.024076755	1.043378022	1.008192251	1.079791772	0.015261497
AC027307.2	0.00093781	1.137822362	1.063699423	1.217110491	0.000172177
PCAT6	0.01287274	1.116752826	1.022680623	1.219478345	0.013913761
AC099850.3	0.044309692	0.93250909	0.886256297	0.981175768	0.007100053
AC011472.1	0.036654682	1.216082131	1.044485384	1.415870219	0.011709428

HR, hazard ratio.

**Table 2:** Multivariate Cox regression analyses of OS in patients with colorectal cancer.

lncRNA	Coef	HR	HR 95 low	HR 95 high	*P value*
SNHG16	-0.899430812	0.40680114	0.212665883	0.778155692	0.006569273
AL590483.1	-0.821055697	0.439966937	0.199861673	0.968524395	0.041409882
ZEB1-AS1	0.914362233	2.495183398	0.958847406	6.493150162	0.060951975
AC107375.1	0.666338657	1.94709527	1.005125512	3.771847343	0.048254771
AC068580.3	0.7681273	2.155725443	0.877008339	5.298868873	0.094138679
AC147067.1	0.557557023	1.746400867	1.005442055	3.033408017	0.047790122

A Cox regression model can be adopted to generate the risk model.

### Establishment of a prognostic risk signature of cell senescence-related lncRNAs in a training dataset

**Figure 3:**
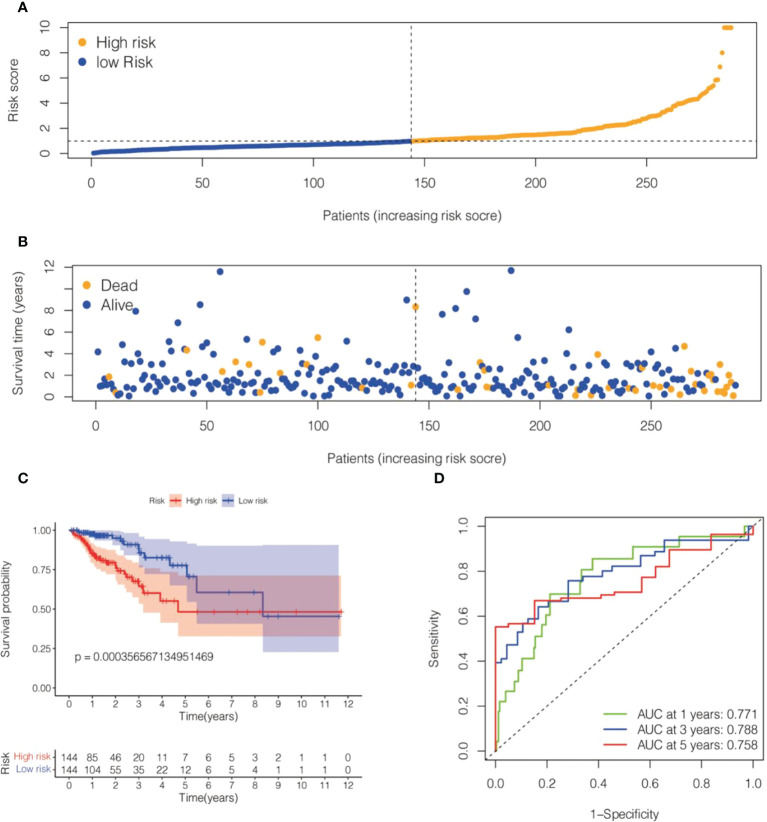
Risk model for 6 cell senescence-related lncRNAs with prognostic value in the TCGA training set. **(A)** Distribution of risk scores and different groupings in the TCGA training set. **(B)** Relationship between risk score and survival status in the TCGA training set. **(C)** Kaplan–Meier survival analysis of the high-risk and low-risk groups in the TCGA training se. **(D)** Time-dependent ROC curve depicting the predictive accuracy of the risk model for OS in the TCGA training set.

We randomly divided TCGA- COADREAD (573) into a training dataset (288) and a validation dataset (285). We calculated the values of 6 cell senescence-related lncRNAs in the training set using the above formula (**Supplementary Table S2**). Patients with CRC in the training dataset were divided into two subgroups (high- and low-risk groups) on the basis of the median risk score. The risk scatterplot showed the risk score calculated by the formula and the survival status (**Figures 3A, B**). Furthermore, Kaplan−Meier curves demonstrated that the low-risk group had a longer survival time than the high-risk group (**Figure 3C**). The ROC curves of the risk model for 1, 3, and 5 years were 0.771, 0.788, and 0.758, respectively (**Figure 3D**).

### Internal validation cohort and GSE17537 dataset to validate the risk model

**Figure 4:**
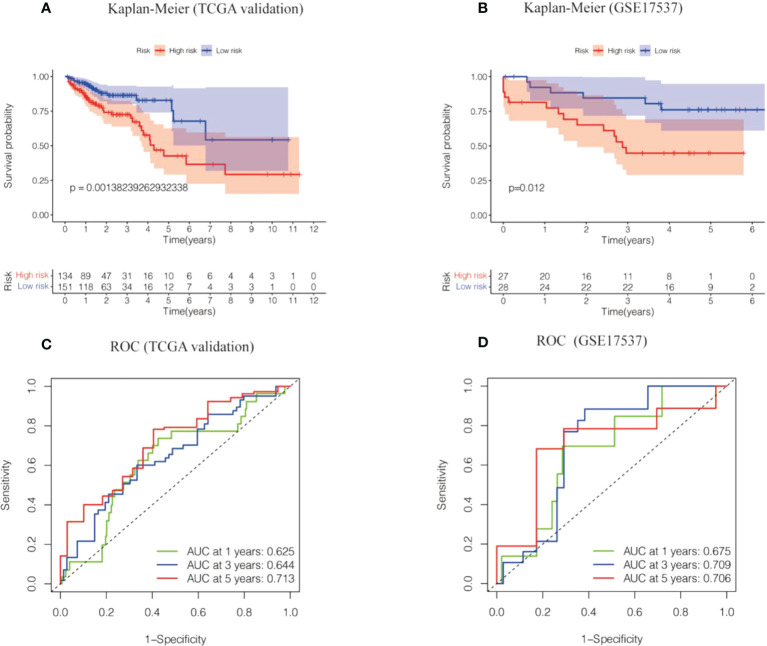
The risk model of the 6 cell cell senescence-related lncRNAs in the TCGA validation cohort and GSE17537. **(A)** Kaplan-Meier analysis of the OS for high and low risk groups in the TCGA validation cohort. **(B)** Kaplan-Meier analysis of the OS for high and low risk groups in GSE17537. **(C)** Time-dependent ROC curve depicting the predictive accuracy of the risk model in the TCGA validation cohort. **(D)** Time-dependent ROC curve depicting the predictive accuracy of the risk model in GSE17537.

Two datasets (the TCGA validation set and the GSE17537 dataset) were used to evaluate the precision and stability of the risk model. Risk scores were generated for each sample on the basis of the risk score formula described above. Patients in the two validation sets were categorized into a high-risk group and a low-risk group based on median risk scores. A Kaplan−Meier analysis indicated that OS was lower in the high-risk group and higher in the low-risk group (**Figures 4A, B**). The ROC values for 1-, 3-, and 5-year survival were 0.7, 0.71, and 0.71 for the TCGA validation dataset and 0.77, 0.74, and 0.75 for the GSE17537 dataset, respectively (**Figures 4C, D**). In conclusion, the prognostic model of the 6-cell senescence-related lncRNA risk score has high accuracy and stability in prognostic assessment. Our prognostic risk model of 6 cell senescence-related lncRNAs more accurately estimated the prognosis of CRC.

### Target genes and functional analysis of the 6 cell senescence-associated lncRNAs

**Figure 5:**
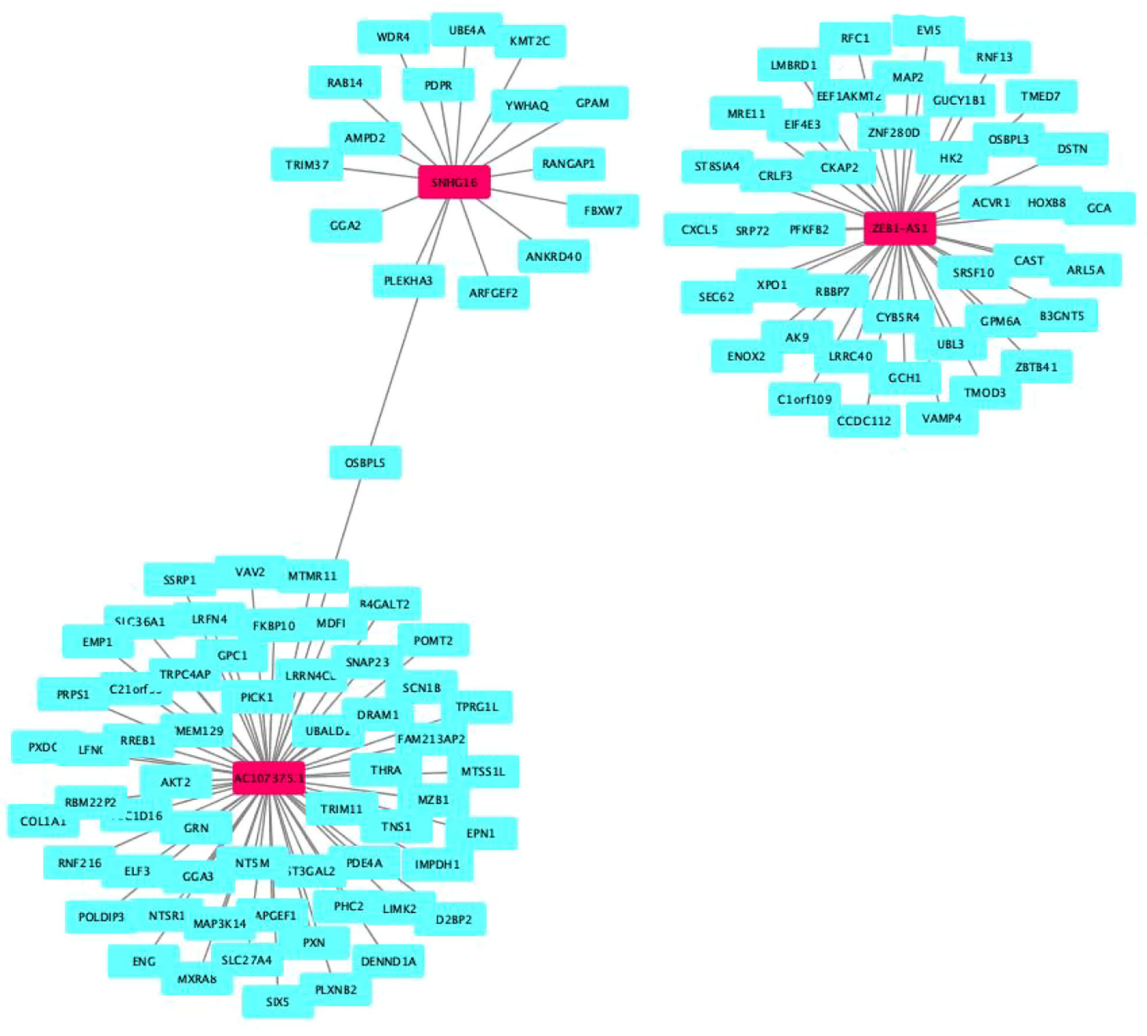
Target genes of cell senescence-related lncRNAs.

**Figure 6:**
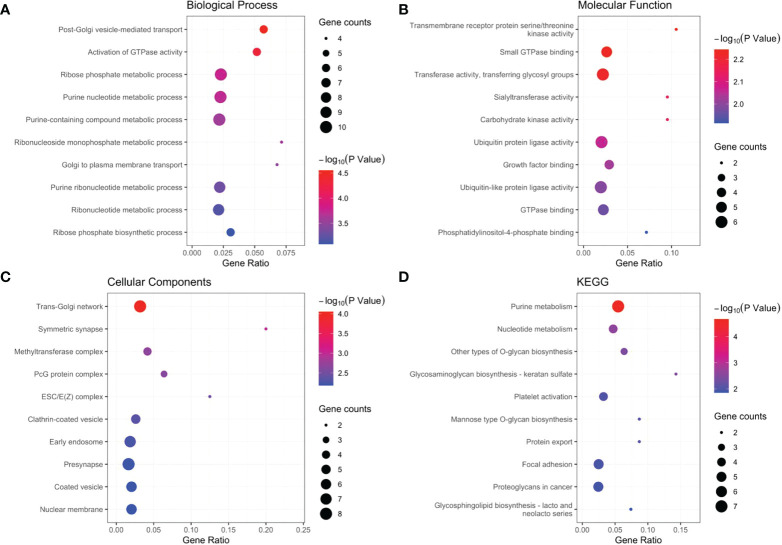
GO and KGEE analysis of the target genes: **(A)** Biological Process (BP) of target genes. **(B)** Molecular Function (MF) of target genes. **(C)** Cellular Components (CC) of target genes. **(D)** KEGG of target genes.

Our results revealed that AL590483.1, AC068580.3 and AC147067.1 did not regulate the coding protein genes; however, SNHG6 may be involved in the regulation of 16 genes (**Supplementary Table S3** and **Figure 5**), ZEB1-AS1 may be involved in the regulation of 43 genes (**Supplementary Table S4** and **Figure 5**) and AC107375.1 may be involved in the regulation of 59 genes (**Supplementary Table S3** and **Figure 5**). The 118 target genes found to be significantly enriched in the biological process terms postGolgi vesicle-mediated transport and activation of GTPase activity (**Figure 6A**). The 118 target genes found to be significantly enriched in the molecular function terms transmembrane receptor protein serine/threonine kinase activity and small GTPase binding (**Figure 6B**). The 118 target genes found to be significantly enriched in the Cellular Component: Trans-Golgi network and symmetric synapse (**Figure 6C**). The 118 target genes found significant enrichment in the KEGG terms purine metabolism and nucleotide metabolism (**Figure 6D**).

### Development of a nomogram and evaluation of the survival model for CRC

**Figure 7:**
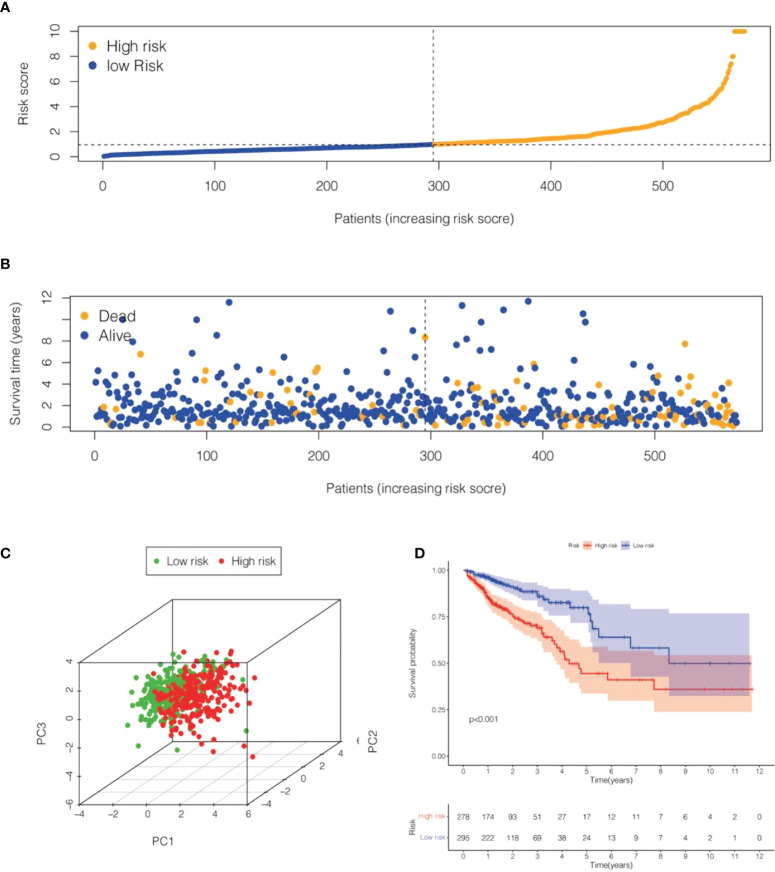
Risk model for 6 cell senescence-related lncRNAs with prognostic value in TCGA-COADREAD. **(A)** Distribution of risk scores and different groupings in TCGA-COADREAD. **(B)** Relationship between risk score and survival status in TCGA-COADREAD. **(C)**. 3D PCA for high and low risk groups in TCGA-COADREAD. **(D)** Kaplan-Meier analysis of the OS for high and low risk groups in TCGA- COADREAD.

**Figure 8:**
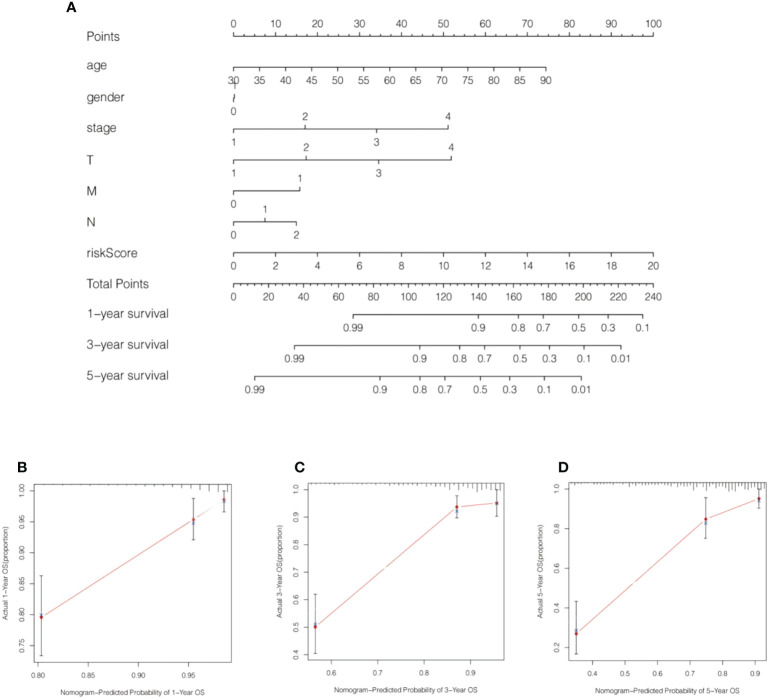
A nomogram was generated by combining risk scores and clinical characteristics. **(A)** A nomogram forecasting 1-, 3-, and 5-year overall survival was constructed. **(B–D)** Calibration curves of the nomogram display the consistency between the predicted and 1-, 3-, and 5-year survival rates.

**Figure 9:**
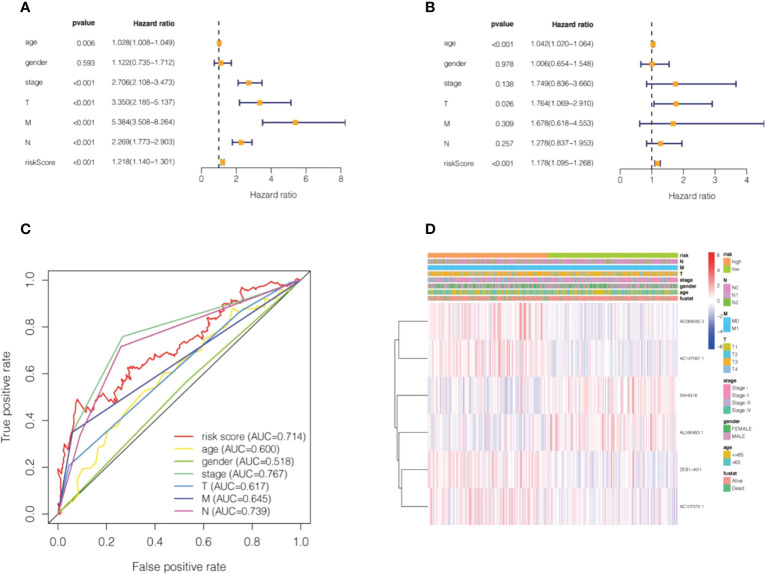
Validation of the risk model of cell senescence-related lncRNAs in colorectal cancer. **(A)** Univariate Cox regression of the risk score and clinical indicators. **(B)** Multivariate Cox regression of the risk score and clinical indicators. **(C)** The AUC value for the risk score and clinical indicators. **(D)** A heatmap of correlations between the risk score and clinical factors.

We calculated the values of 6 cell senescence-related lncRNAs in TCGA- COADREAD (573) using the above formula. On the basis of the median expression value of 6 cell senescence-related lncRNAs, we classified them into high- and low-risk groups. The risk scatterplot displayed the risk score calculated by the risk formula and the survival status (**Figures 7A, B**). 3D PCA showed that patients with various risk scores were divided into two clusters (**Figure 7C**). Furthermore, we performed Kaplan−Meier analysis on the basis of the high- and low-risk groups (**Figure 7D**). We constructed a nomogram to further forecast patient outcomes. The results showed that the distribution of different clinical index values and risk score values for CRC had different degrees of contribution throughout the scoring process (**Figure 8A**). We performed a predictive analysis of 1-, 3-, and 5-year OS, showing good predictive power of the nomogram (**Figures 8B–D**). Calibration curves were used to test the consistency between actual OS rates and predicted survival rates at 1, 3, and 5 years. To assess whether our risk model was an indicator of independent prognosis in CRC, we also performed univariate and multivariate Cox regression analyses. Our current findings suggested a hazard ratio (HR) of 1.218 (95% CI 1.140-1.301) (*P*< 0.001) for risk scores in univariate Cox regression analysis (**Figure 9A**) and a hazard ratio (HR) of 1.178 (95% CI 1.095-1.268) for risk scores in multivariate Cox regression analysis (*P*< 0.001). (**Figure 9B**). In conclusion, the risk model is an independent prognostic factor for CRC. The AUC point of the risk score was 0.714, indicating that the risk model was a robust prognostic risk model for CRC (**Figure 9C**). A heatmap of correlations between prognostic signatures of cell senescence-related lncRNAs and clinicopathological outcomes was also generated. According to the heatmap, the clinical features showed no significant differences (**Figure 9D**). Finally, we explored the relationship between the clinical features and 6 cell senescence-related lncRNAs. ZEB1-AS1 expression correlated with age, sex, TNM stage and stage (**Supplementary Figure S1**). AC068580.3 expression did not correlate with clinical features (**Supplementary Figure S2**). AC107375.1 expression correlated with stage, N stage and M stage (**Supplementary Figure S3**). AC147067.1 expression correlated with stage and T stage (**Supplementary Figure S4**). AL590483.1 expression did not correlate with clinical features (**Supplementary Figure S5**). SNHG16 expression correlated with stage and N stage (**Supplementary Figure S6**).

### Immune-related multiomics analysis

**Figure 10:**
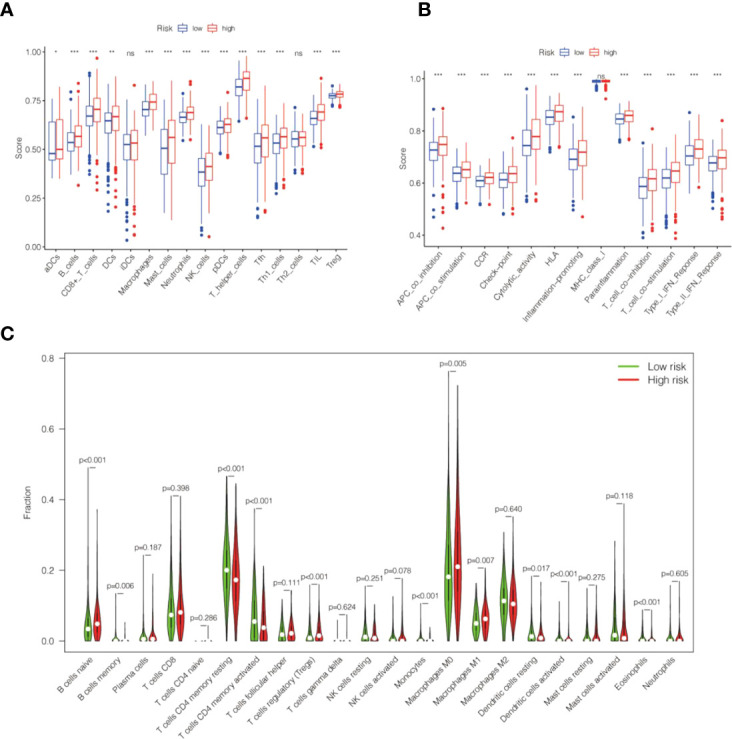
The correlation between the risk score of the cell senescence-related lncRNAs and tumor infiltrate. **(A)** Box plot revealing the relationship between immune cells and the risk score. **(B)** The box plot demonstrates the correlation between immune function and risk score. **(C)** Violin plot indicating the 22 tumor-infiltrating lymph cell distributions with different risk scores. *P<0.05, **P<0.01, ***P<0.001 and NS>0.05.

We explored the relevance of risk scores and immune function and immune cells. We used ssGSEA to calculate different immune cells and immune function scores according to the gene expression levels of each sample. The results indicated that immature dendritic cells (iDCs) and T helper type 2 (Th2) cells did not differ remarkably between the high-risk and low-risk groups (**Figure 10A**). The immune cell scores of the high-risk group, including B cells, activated dendritic cells (aDCs), CD8+ T cells, dendritic cells (DCs), macrophages, immature dendritic cells (iDCs), mast cells, neutrophils, NK cells, plasmacytoid dendritic cells (pDCs), T helper cells, T follicular helper (Tfh), Th1 cells, tumor-infiltrating lymphocytes (TILs), and T regulatory cells (Tregs), were significantly higher than those of the low-risk group, and the difference was statistically significant (**Figure 10A**).

Regarding immune function, our findings revealed that the grades of the high-risk group were markedly higher than those of the low-risk group, including antigen-presenting cell (APC) cosuppression, APC costimulation, chemokine receptor (CCR), checkpoints, cytolytic activity, human leukocyte antigen (HLA), inflammation promotion, para-inflammation, T-cell cosuppression, T-cell cosuppression of stimulation scores, type I immune response, and IFN response (**Figure 10B**).

**Figure 11:**
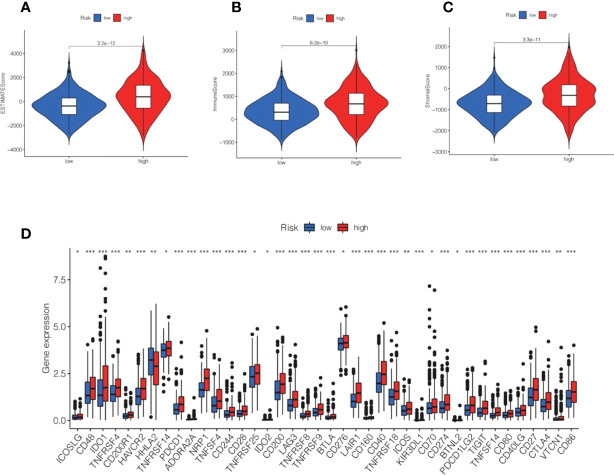
Correlation between ESTIMATE and immune checkpoint and risk score. **(A–C)** Violin shows the relationship between ESTIMATE and different subgroups. **(D)** Box plot displaying the correlation between the risk score and immune checkpoint.*P<0.05, **P<0.01, ***P<0.001.

CIBERSORT analysis suggested that naive B cells, regulatory T cells, M0 macrophages, M1 macrophages, and activated dendritic cells were markedly higher in the high-risk group. In contrast, resting memory CD4 T cells, activated memory CD4 T cells, monocytes, resting dendritic cells, and eosinophils were markedly reduced in the high-risk group (**Figure 10C**). Further analysis of the low-risk group showed markedly lower ESTIMATEScore, ImmuneScore, and StromalScore (**Figures 11A–C**).

Immune checkpoint analysis showed that the immune checkpoint gene expression we detected was higher in the high-risk group than in the low-risk group, and there was also a statistically considerable difference. (**Figure 11D**). The level of PD-L1 (CD274) was significantly increased in high-risk patients, revealing that high-risk groups were far more prone to respond to anti-immunotherapy than low-risk groups (**Figure 11D**).

### Drug sensitivity analysis

**Figure 12:**
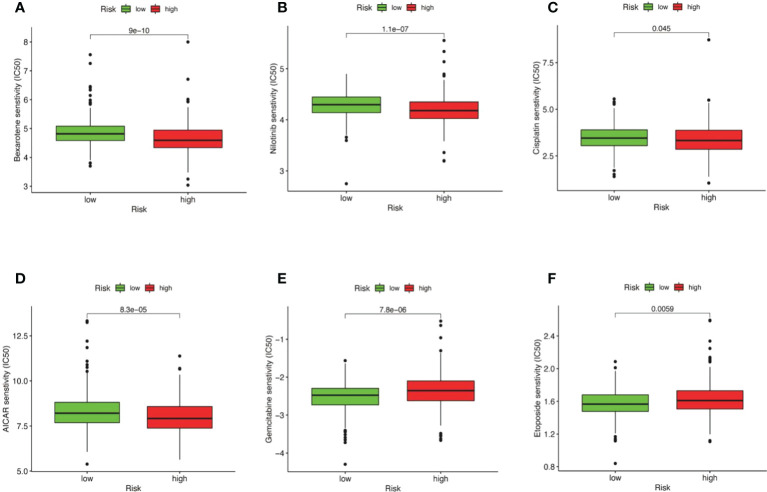
Correlation between risk score and drug resistance. The box plot displays the relationship between the risk score and the IC50 of various drugs. **(A)** IC50 of bexarotene in high and low risk groups. **(B)** IC50 of nilotinib in high and low risk groups nilotinib. **(C)** IC50 of cisplatin in high and low risk groups. **(D)** IC50 of AICAR in high and low risk groups. **(E)** IC50 of Gemcitabine in high and low risk groups. **(F)** IC50 of etoposide in high and low risk groups.

We established an association between the risk score and chemotherapy response in CRC by GDSC. The results indicated that bexarotene, nilotinib, cisplatin, and AICAR exhibited lower IC50 values in the high-risk group (**Figures 12A–D**). Gemcitabine and etoposide exhibited substantially higher IC50 values in the high-risk group (**Figures 12E, F**). The IC50 and AUC of the above drugs on CRC cell lines are shown in **Supplementary Table S6**.

## Discussion

The incidence of CRC is increasing year by year (42). There are many therapeutic strategies for CRC, including surgery, immunotherapy, radiotherapy, and targeted therapy, but the treatment outcome is still dismal (43). TNM staging based on tumor size, number of lymph nodes, and metastasis can no longer satisfy the prognostic prediction of malignant colorectal tumors and the guidance of more accurate treatment. There is an urgent need to explore potential biomarkers and effective treatment prediction methods. Currently, numerous investigators have focused on the critical roles of lncRNAs in the cellular senescence of malignant tumors. In the past two years, an increasing number of investigations have focused on the prognostic prediction of malignancy by lncRNA signatures, such as gastric cancer and CRC (44–46). Zhiwei Wu et al. found that the establishment of a risk model with four ferroptosis-related lncRNAs has many predictors of prognosis in patients with colon cancer (47). Lili Li et al. found that the m6A methyltransferase-related lncRNA signature could forecast the prognosis and immunotherapy response of liver cancer patients (48). However, the signatures of cell senescence-related lncRNAs in CRC have not been elucidated. In our study, a coexpression network of cell senescence-related mRNA−lncRNA was explored by R. Six cell senescence-related lncRNA signatures were identified using univariate and multivariate analyses. The risk model was created by six cell senescence-related lncRNAs, including SNHG16, AL590483.1, ZEB1-AS1, AC107375.1, AC068580.3, and AC147067.1. Fengfeng Xu et al. found that SNHG16, which might serve as a new tumor suppressor, was downregulated in liver cancer cells and could inhibit the proliferation of liver cancer cells by sponging hsa-mir-93 (49). ZEB1-AS1 promoted tumorigenesis and progression. Guohua Wei et al. found that ZEB1-AS1 enhanced tumor progression through upregulation of TRIB2 expression (50). Consistent with our study, SNHG6 serves as a tumor suppressor, and ZEB1-AS1 acts as a tumor promoter. To validate the robustness and reliability of the model, we adopted TCGA internal verification and GSE17537 for external verification. The ROC values for 1-, 3-, and 5-year survival were 0.7, 0.71, and 0.71 for the TCGA validation set and 0.77, 0.74, and 0.75 for the GSE17537 dataset, respectively. The results show that the risk model has good stability and accuracy.

Malignant tumor tissues consist not only of tumor cells but also normal tumor epithelial cells, stromal cells, immune cells, and vascular cells. More importantly, stromal cells are significantly correlated with tumor growth and tumor resistance. Mesenchymal stromal cells can promote tumor metastasis and EMT polarization through M2 macrophages (51). Our current research revealed that the low-risk group had lower ESTIMATESocre, ImmuneScore, and StromalScore. Recent studies have also indicated that cell senescence is intimately linked with tumor immunity. A study strongly suggested that the secretion of SASP by senescent endothelial cells promotes the invasive behavior of tumor cells *via* CXCL11 targeting the tumor microenvironment (52). Similarly, A. Krtolica et al. indicated that senescent fibroblasts led to tumorigenesis by premalignant and malignant epithelial cells (53). Furthermore, the potential effects of interleukins in SASP on the tissue microenvironment can induce the ability of epithelial-mesenchymal transition (EMT) to stimulate tumor progression (54). Our current study revealed that cell senescence-related lncRNA prognostic signatures are intimately associated with the TME and are strongly related to the outcome of patients with CRC. CIBERSORT analysis suggested that naive B cells, regulatory T cells, M0 macrophages, M1 macrophages, and activated dendritic cells were markedly higher in the high-risk group. Based on ssGSEA, B cells, activated aDCs, CD8+ T cells, DCs, macrophages, iDCs, mast cells, neutrophils, NK cells, pDCs, T helper cells, Tfh cells, Th1 cells, TILs, and Tregs were considerably higher in the high-risk group. CD4+ regulatory T (Treg) cells are a highly immunosuppressive subset that impedes immune surveillance of cancers, such as CRC, breast malignant tumors, and gastric cancer (55–57). These findings may provide new ideas for understanding characteristic immune cells and our risk score model. Immune checkpoint molecules refer to ligand−receptor pairs that inhibit or stimulate immune responses. Immune checkpoints play important immunomodulatory roles in maintaining immune homeostasis and preventing autoimmunity (58). Currently, the most extensively studied inhibitory immune checkpoint routes consist of CTLA-4, PD-1 (CD274), and PD-L1 (59, 60). The levels of PD-L1 (CD274) and CTLA-4 were significantly increased in high-risk patients. Our findings implied that the high-risk group could respond to anti-PD-L1 or CTLA-4 immunotherapy and may act as a promising predictive tool to forecast the response to anti-immune drugs.

There are many treatment methods for CRC. Chemotherapy is one of the most important methods of treatment (61). However, chemotherapy drugs have responded differently to different patients. Recently, there has been considerable enthusiasm for the establishment of tumor drug sensitivity prediction models and computational methods for the more precise use of chemotherapy drugs by individuals (61). Accurately predicting an individual’s drug susceptibility is a challenging task. The results indicated that AICAR, cisplatin, nilotinib, and bexarotene exhibited lower IC50 values in the high-risk group. Etoposide and gemcitabine were higher in the high-risk group. However, AICAR and bexarotene are not used in the clinical management of CRC. However, elaboration of the association between risk models of cell senescence-related lncRNAs and drug sensitivity may reveal the potential role of these drugs in the treatment of CRC. Our molecular stratification of CRC patients based on risk scores to predict drug sensitivity may optimize tumor chemotherapy.

Our current study was the first to utilize bioinformatics analysis of cell senescence-related lncRNAs in CRC, and there are still some drawbacks. First, a cell senescence-related lncRNA risk model was used to explore immune infiltration, immune checkpoints, and drug susceptibility analysis without *in vitro* or *in vivo* experiments. Therefore, the clinical use of this risk model needs to be further elucidated. Furthermore, accurate prediction of CRC prognosis is challenging and may be associated with high heterogeneity of the tumor. Specifically, we adopted only genes associated with cell senescence to generate prognostic risk models, and other critical genes may not be included. In any case, our risk model has the potential to act as a prognostic biomarker, providing unique insights for CRC.

## Conclusion

Our current study identified 6 cell senescence-associated lncRNA signatures that may be vital biomarkers to predict the prognostic features and immune and chemotherapy responses in CRC.

## Data availability statement

The original contributions presented in the study are included in the article/**Supplementary Material**. Further inquiries can be directed to the corresponding authors.

## Author Contributions

JX, CY, and CX proposed the conception and implementation of the paper. ZL and FL were responsible for data download and analysis and participated in the preparation of the manuscript. JX and CY performed the final proofreading. All authors read and approved the final manuscript.

## Funding

The Key Project of Sichuan Provincial Department of Science and Technology Applied Basic Research Program, China (NO. 2022NSFSC0050). The Key project of the First Affiliated Hospital of Chengdu Medical College (NO. CYFY2018ZD03: CYFY-GQ17). National Natural Science Foundation of the P.R. of China (No.81070378). Sichuan Outstanding Youth Fund Project Grant ( No. 215JQ0060).

## Acknowledgments

The authors acknowledge Dr. Zhang Shihai for providing R language.

## Conflict of interest

The authors declare that the research was conducted in the absence of any commercial or financial relationships that could be construed as a potential conflict of interest.

## Publisher’s note

All claims expressed in this article are solely those of the authors and do not necessarily represent those of their affiliated organizations, or those of the publisher, the editors and the reviewers. Any product that may be evaluated in this article, or claim that may be made by its manufacturer, is not guaranteed or endorsed by the publisher.

## Supplementary material

Supplementary Table 1 1161 cell senescence-related lncRNAs in TCGA-COADREAD.

Supplementary Table 2 The values of 6 cell senescence-related lncRNAs in the training set.

Supplementary Table 3 SNHG6 is involved in the regulation of 16 genes.

Supplementary Table 4 ZEB1-AS1 is involved in the regulation of 43 genes.

Supplementary Table 5 AC107375.1 is involved in the regulation of 59 genes.

Supplementary Table 6 The IC50 and AUC of the drugs on CRC cell lines.

Supplementary Figure 1 Relationship between ZEB1-AS1 expression and clinical features.

Supplementary Figure 2 Relationship between AC068580.3 expression and clinical features.

Supplementary Figure 3 Relationship between AC107375.1 expression and clinical features.

Supplementary Figure 4 Relationship between AC147067.1 expression and clinical features.

Supplementary Figure 5 Relationship between AL590483.1 expression and clinical features.

Supplementary Figure 6 Relationship between SNHG16 expression and clinical features.

Supplementary Code S1 The code for the R language data analysis in the paper.
