# Supplementary figures and images for: A novel cell senescence-related IncRNA survival model associated with the tumor immune environment in colorectal cancer
Source: Front Immunol. 2022 Oct 6;13:1019764. doi: 10.3389/fimmu.2022.1019764 (PMC9583265; doi:10.3389/fimmu.2022.1019764)

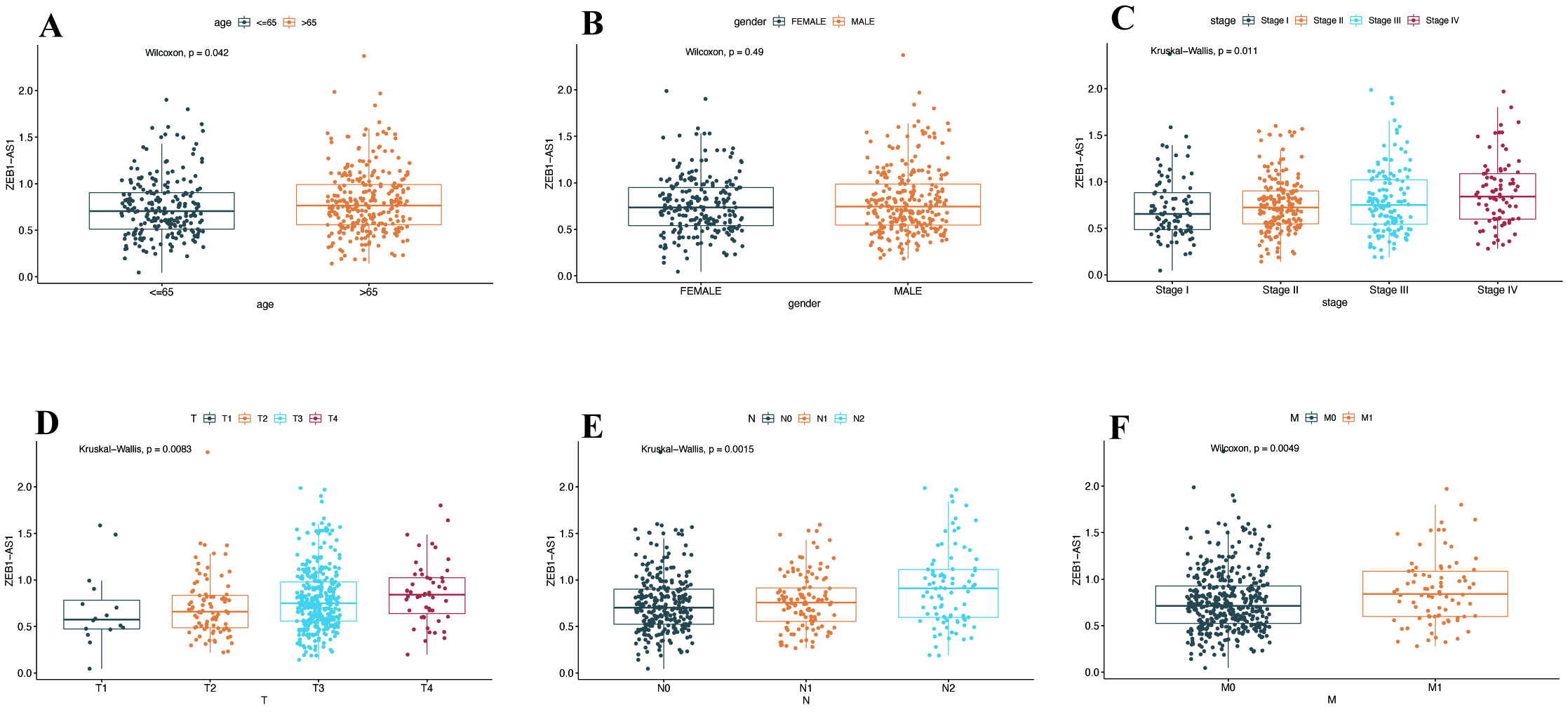

Supplement: Supplementary Table 1 — 1161 cell senescence-related lncRNAs in TCGA-COADREAD. [file DataSheet_1.zip › supplementary/Supplementary Figure S1.tif]

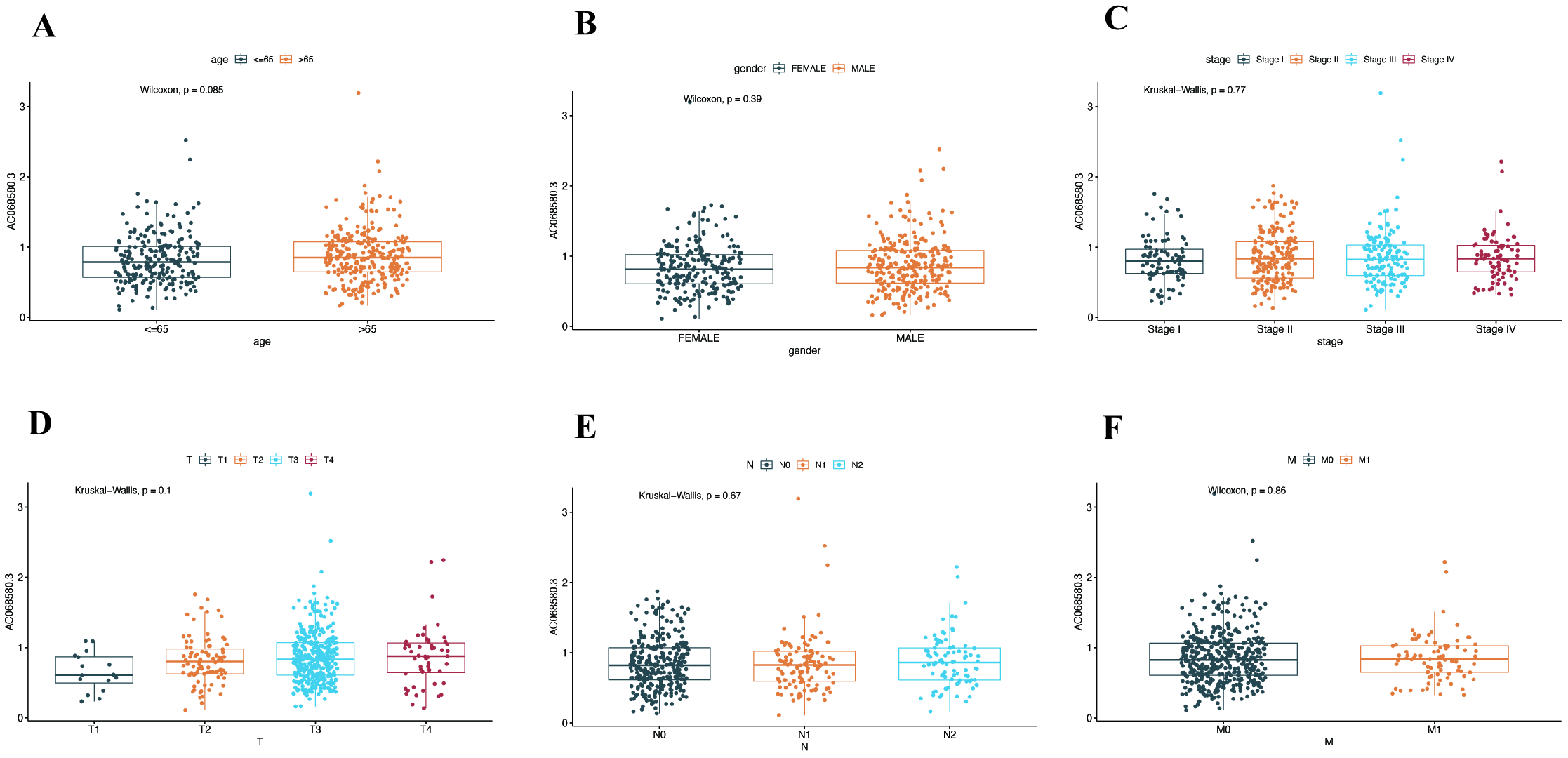

Supplement: Supplementary Table 1 — 1161 cell senescence-related lncRNAs in TCGA-COADREAD. [file DataSheet_1.zip › supplementary/Supplementary Figure S2.tif]

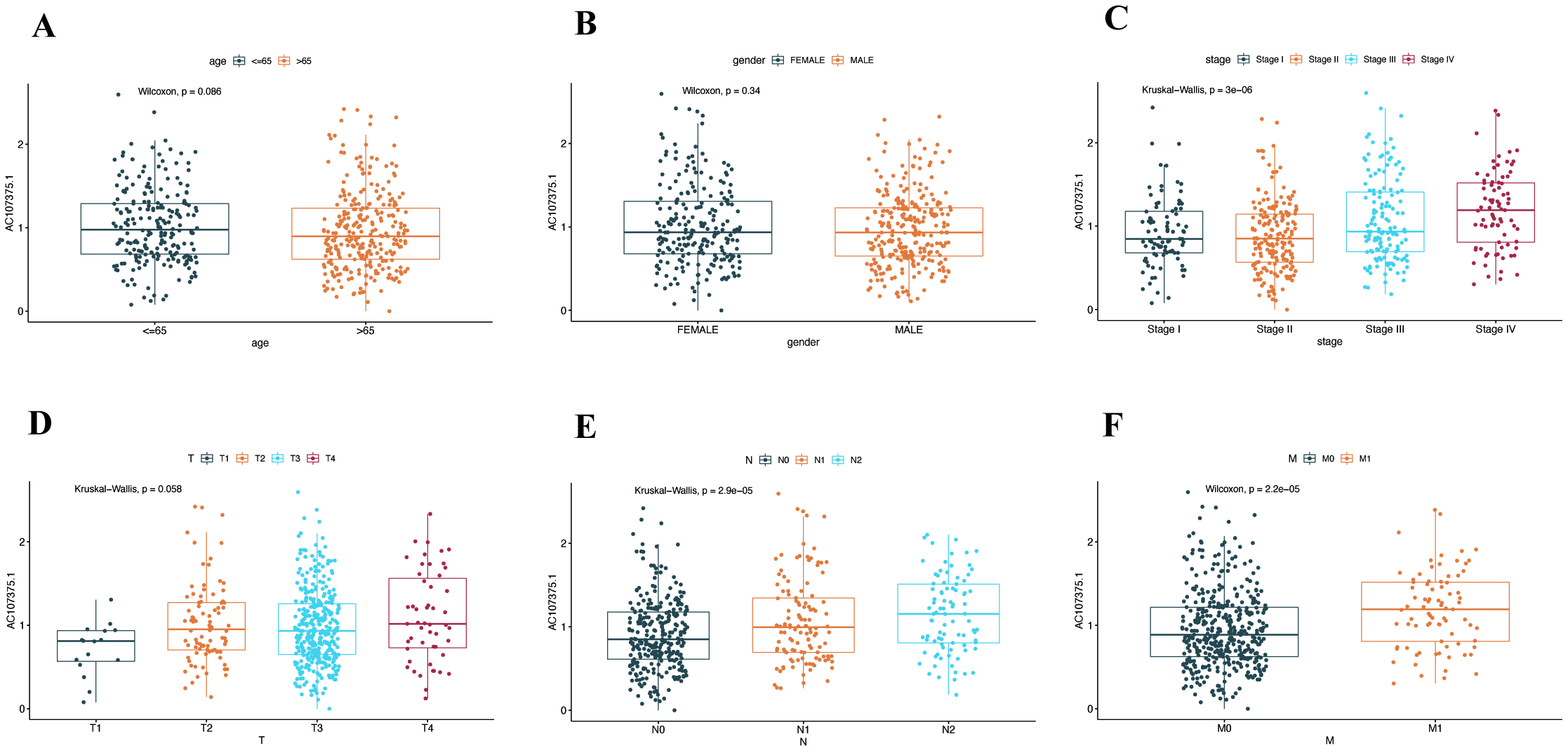

Supplement: Supplementary Table 1 — 1161 cell senescence-related lncRNAs in TCGA-COADREAD. [file DataSheet_1.zip › supplementary/Supplementary Figure S3.tif]

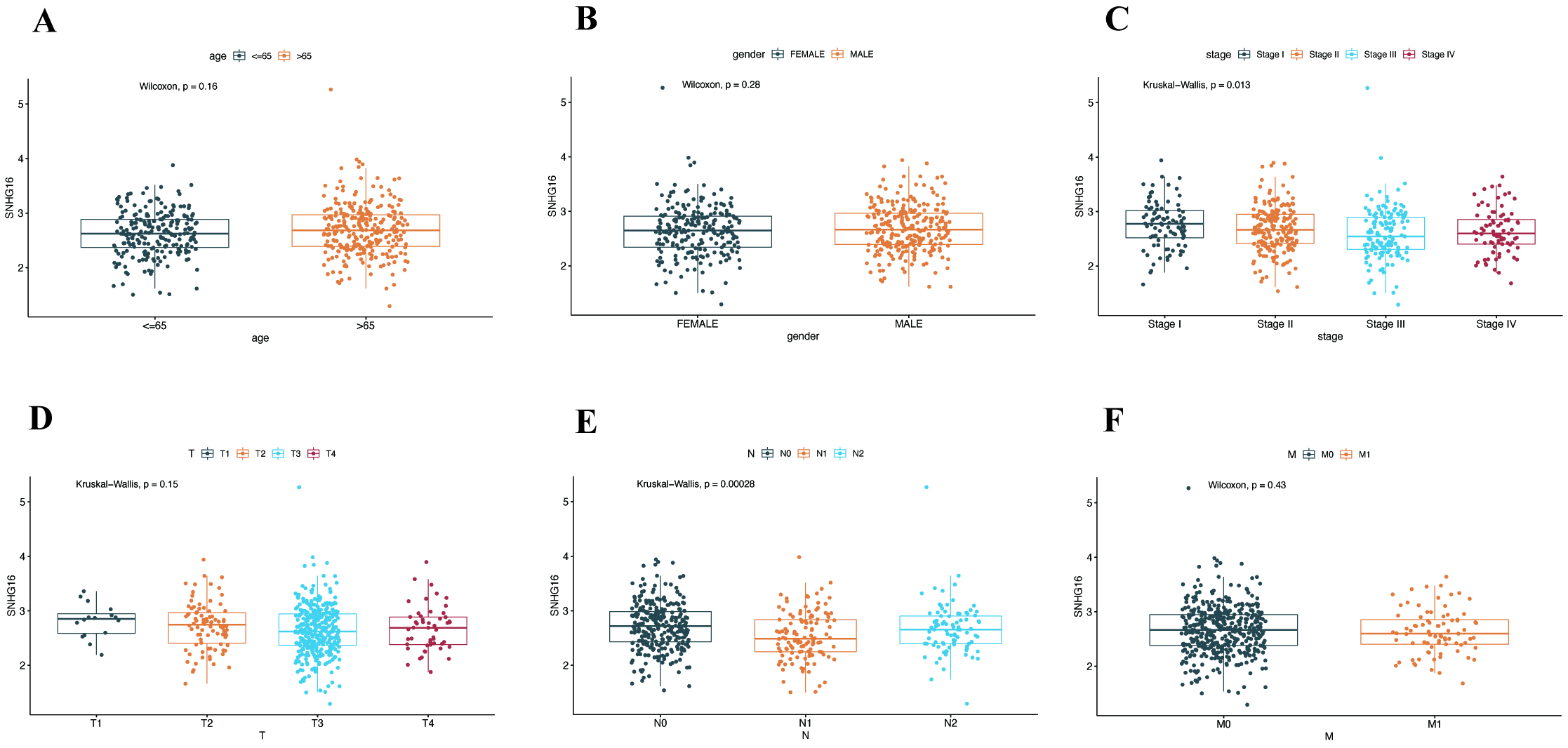

Supplement: Supplementary Table 1 — 1161 cell senescence-related lncRNAs in TCGA-COADREAD. [file DataSheet_1.zip › supplementary/Supplementary Figure S6.tif]

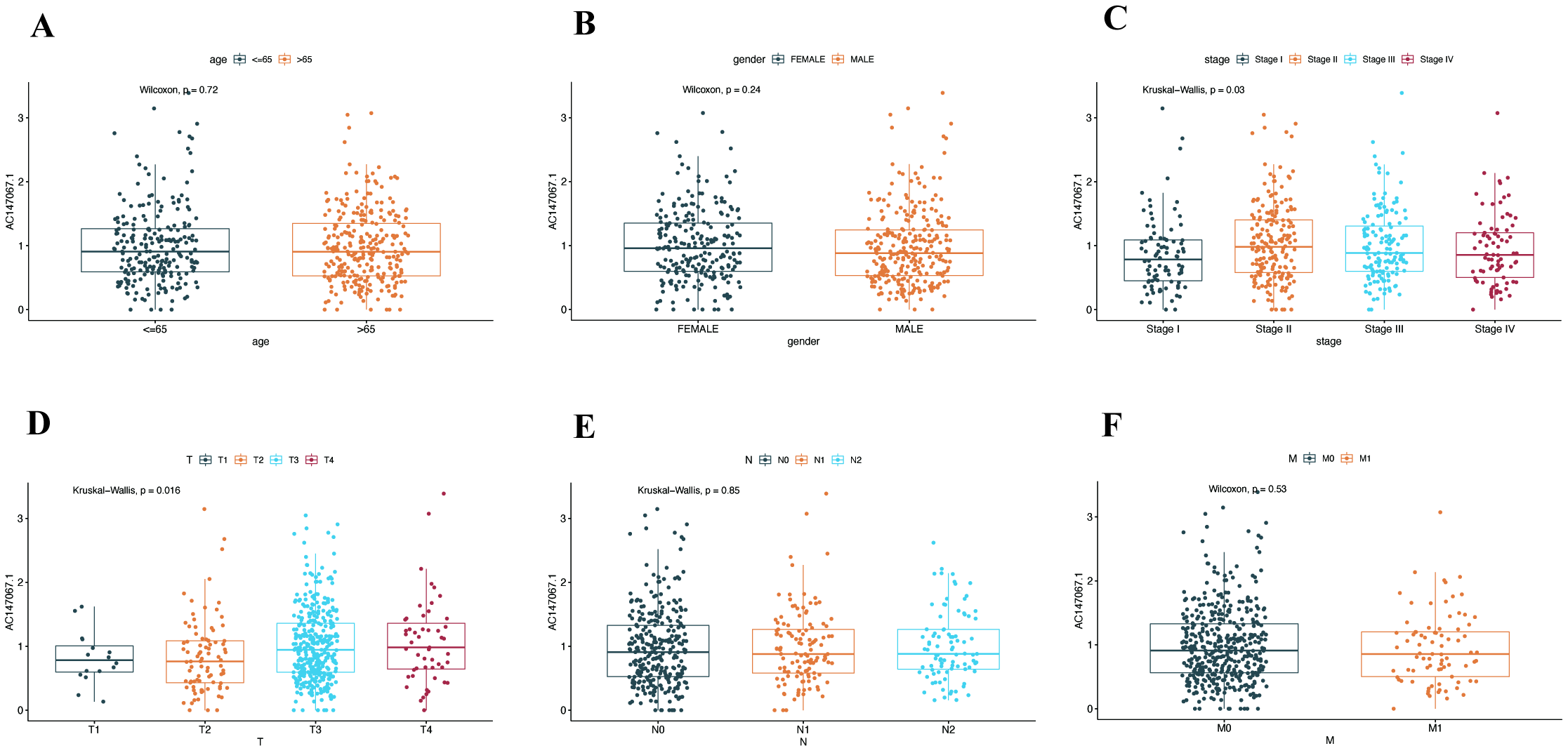

Supplement: Supplementary Table 1 — 1161 cell senescence-related lncRNAs in TCGA-COADREAD. [file DataSheet_1.zip › supplementary/Supplementary Figure S4.tif]

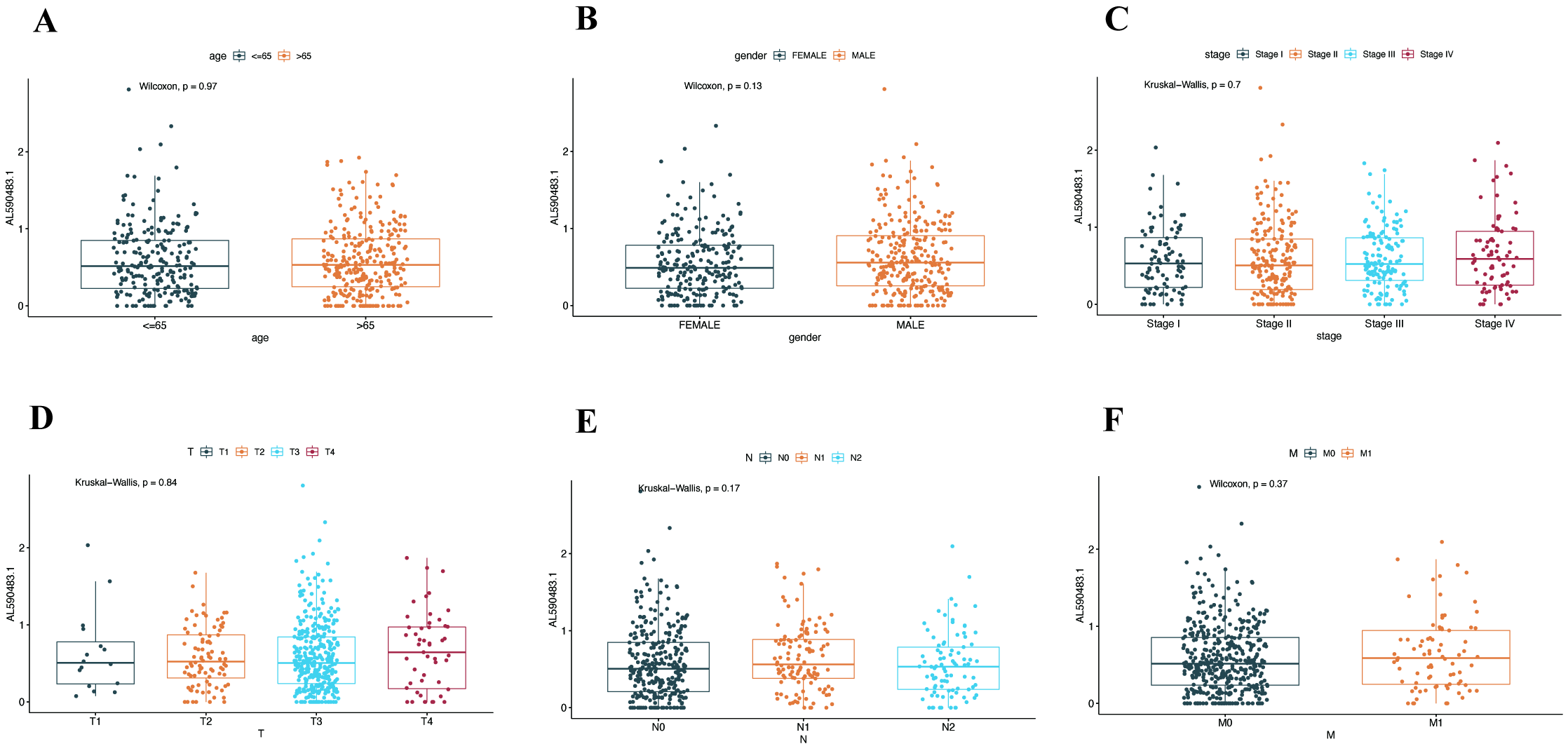

Supplement: Supplementary Table 1 — 1161 cell senescence-related lncRNAs in TCGA-COADREAD. [file DataSheet_1.zip › supplementary/Supplementary Figure S5.tif]
